# Testing optimal methods to compare horse postures using geometric morphometrics

**DOI:** 10.1371/journal.pone.0204208

**Published:** 2018-10-31

**Authors:** Emilie Sénèque, Stéphane Morisset, Clémence Lesimple, Martine Hausberger

**Affiliations:** 1 UMR 6552 Ethologie Animale et Humaine, CNRS, Université de Rennes, Université de Caen-Normandie, Rennes, France; 2 Stéphane Morisset Independent biostatistician, Pérouges, France; 3 CNRS, UMR 6552 Ethologie animale et humaine, Université de Rennes, Université de Caen-Normandie, Rennes, France; University of Illinois, UNITED STATES

## Abstract

The study of animal behavior, especially regarding welfare, needs the development of tools to identify, quantify and compare animal postures with interobserver reliability. While most studies subjectively describe animal postures, or quantify only limited parts of the body, the usage of geometric morphometrics has allowed for the description of horses’ and pigs’ upper body outline and the comparison of postures from different populations thanks to robust statistical analysis. We have attempted here to optimize the geometric morphometrics (GM) method already used in horses by introducing the outline analysis with sliding semilandmarks (SSL), by eliminating the balance movement of the neck and by focusing only on parts of the upper line. For this purpose, photographs of 85 horses from 11 riding schools, known for differing in terms of housing and working conditions, were analyzed with previous and new GM methods and these results were compared with each other. Using SSL and eliminating the neck movement appeared to better discriminate the horse populations than the previous GM method. Study of parts of the dorsum proved efficient too. This new methodology should now be used to examine if posture could be an indicator of horse welfare state, and similar studies should be performed in other species in order to validate the same methodology.

## Introduction

Precise description of animal postures is fundamental for using it as a possible indicator of emotional or welfare state [1–3]. Most studies of global postures rely upon subjective comparisons of individual postures and have therefore not been quantitative [1, 4–6]. Some objective methods are used to compare postures, but then body postures are often described using only some parts of the body: ears, tail, body (e.g. [3, 7–10]); or the general position of the body in space [4–6, 11]. Other studies have used measures of angles between different parts of the body [12, 13]. Kinematic studies using measures of angles between markers stuck or painted on animals, or surgically implanted (e.g. horses: [14–18]; ferrets: [19–21]), allow quantitative measures of the global posture. However these methods are constraining as animals have to be maintained in standardized conditions (hence their postures are not spontaneous) and require the use of very expensive, high quality and specific recording and processing material. Furthermore, these methods of angle measurement or kinematic approaches bring an important amount of data that are not easy to analyze.

A new quantitative method, based on geometric morphometrics (GM) modeling has been recently developed for describing postures, thanks to markers on the back, neck and head of horses [22] and pigs [23]. GM analyzes the relative positions of landmarks (i.e. anatomical points which are homologous between subjects) in order to model forms, curves (outlines) or surfaces. Generalized Least Squares (GLS) method, also called Procrustes Superimposition, extracts the geometric information required to quantify differences in shape, regardless of size, orientation and position of the subject [24]. Geometric information can then be treated by standard and robust statistical analysis [25]. In addition, all the landmark configurations can be rendered through a Principal Component Analysis representation (PCA) where the consensus configuration (i.e. the mean configuration) is the axis origin. Shape differences can be visualized using the thin-plate spline method that provides vector visualizations or deformation grids [26]. Thus GM provides methods enabling us to compare both subjects or populations, while ensuring interobserver reliability.

Landmarks (LD) located along the dorsum of the horse (from tail to middle of the head) were used in [22]. Data acquisition and treatment were very simple: postures were captured by taking photographs which were then analyzed (i.e. LD drawing, GLS and statistical analyzes) with free softwares (i.e. tps softwares). Modeling the dorsum of the horses with GM enabled us to identify groups of postures according to behaviour (walking, standing, etc.) or conditions of life. In particular, it was found that horses living in more natural conditions had rounder postures, especially at the neck level [22].

The present study goes further by modeling more precisely and more extensively the dorsal outline of the horse. This structure can actually be considered as a homologous curve between subjects, and can thus be accurately described and studied with points that don’t need to be homologous [27], i.e. sliding semilandmarks [28], in addition to landmarks. The principle is to draw a curve with an important number of arbitrarily placed semilandmarks and then to optimize the position of each semilandmark by allowing them to slide along the curve. More precisely, this procedure adds a step on the standard Procrustes Superimposition: besides translation, scaling and rotation of landmarks to optimally improve their superimposition (like for landmarks), the semilandmarks can be slid along the curve in order to make them match the position of the corresponding points in the average curve of the entire sample as well as possible. This step removes the effect of the arbitrary initial spacing of points along the curve. Semilandmarks and landmarks can then be treated the same way.

Furthermore, the neck movement is a major source of shape deformation [22]. Beside this, other variations of shape contributing to posture are less visible in the deformation grids. So it appeared interesting to be able to study the posture in movement without the interference of the neck movement (considered here as a rotation of the neck in relation to the rest of the body in the sagittal plane). Eliminating this rotation, using a fixed angle between the croup/back and the neck/head [29], can be advantageous to optimize the visualization of the difference of shape, by limiting the Pinocchio effect [30]. The Pinocchio effect occurs for example when two sets of landmarks, bound together by a common landmark, move in relation to one another. The generalized least-squares algorithm attemps to minimize the overall difference of configuration by reducing variations between the two sets of landmarks, leading to an increase in the variation around the common and relative fixed landmarks on the deformation grids [31]. The Pinocchio effect disturbs the visualization of the difference of shape within the deformation grids, but it doesn’t impact on the statistical analysis.

Investigating the upper line, combining back, neck and head posture is particularly interesting. Considering horses, several studies indicate that stress, strong working constraints and inappropriate riding techniques or equipment (i.e. saddle and bit) can induce chronic postures (*e*.*g*. a hollow back) [32–36] and back pain [37–40]. According to data in the literature, all authors agree that horses' back problems are very frequent: several studies on riding school horses found that 49,7% to 88% of the individuals were suffering from back disorders [38, 39, 41, 42]. According to Jeffcott et al. [43], back pain in horses is one of the most common and least understood problems in sport horses. Several authors have highlighted that back disorders were a major cause of poor performance [44–47]. Some studies on racehorses and sport horses suggest an impact of sex, age and breed on the prevalence of back lesions, but this is still very controversial [39, 45, 48]. Moreover, in riding school horses, chronic body posture has been shown to reflect overall life conditions (including type of work, [22]) as well as the vertebral state of horses [42], independently of breed or age. This shows that the study of horse posture could be a valuable tool for evaluating their welfare. In the present study, we tried to identify the best method amongst different GM approaches to optimize the characterization of different riding school horse populations. Differences in management have a large impact on horses’ welfare, amongst which are the riding techniques [48]. This methodological study is a first step towards identifying the link between management practices, welfare indicators and horse posture as a potential indicator.

## Material and methods

Experiments complied with current French laws (Centre National de la Recherche Scientifique) related to animal experimentation and were in accordance with the European directive 86/609/CEE. No license/permit/institutional ethical approval was needed. Animal subjects were not exposed to distressing conditions during the study. Animal husbandry and care were under the management of riding school staff. Riding schools' managements authorized the experimenters to conduct their research. This experiment involved only horses in the “field” (no laboratory animals).

### Horses

This study was performed on 85 horses (50 geldings and 35 mares) from 11 riding schools (the median per riding schools is 8 horses, ranging from 1 to 13 horses). They worked in riding lessons involving children and teenagers for 4–14 hours per week, with at least one free day per week. They were only used for teaching, with riders from beginner to intermediate levels.

Official identification documents record the sex, age and breed of horses. They belonged to 9 different breeds (N = 48 horses) or were unregistered (N = 37), which prevented us from testing for a potential breed effect. However, since other studies [37,49, 50] have observed differences between types of equids (e.g. pony / horses, “warmbloods”/ “coldbloods”) the animals were divided into two “classical” official types: pony (<1.48m high at the withers, International Federation for Equestrian Sport) or horse (>1.48m high at the withers) in the analysis.

The horses’ age and sex distribution did not differ between schools (respectively Kruskal-Wallis, p = 0,075; and chi-squared, p = 0,524). The mean age is 13 (7–20 year old).

Using the parameters described in several studies [51–53], we further classified the animals into 3 categories, based on their **proportions**: dolichomorphic (length > height, ex: thoroughbred, purebred Arab), mesomorphic (length = height, ex: French saddlebred) and brachymorphic (length < height, ex: Merens horses).

Horses were under the management of riding schools, mostly housed in straw-bedded individual stalls (91,8%) or in groups in pastures (8,2%). Most of them (87,1%) were fed industrial pellets, once (21,2%), twice (42,4%) or three times (23,5%) per day. All horses received hay in one (52,9%), two (23,5%) or three (15,3%) meals or ad libitum (8,2%). All horses had water ad libitum.

### Data recording

**Fig 1:**
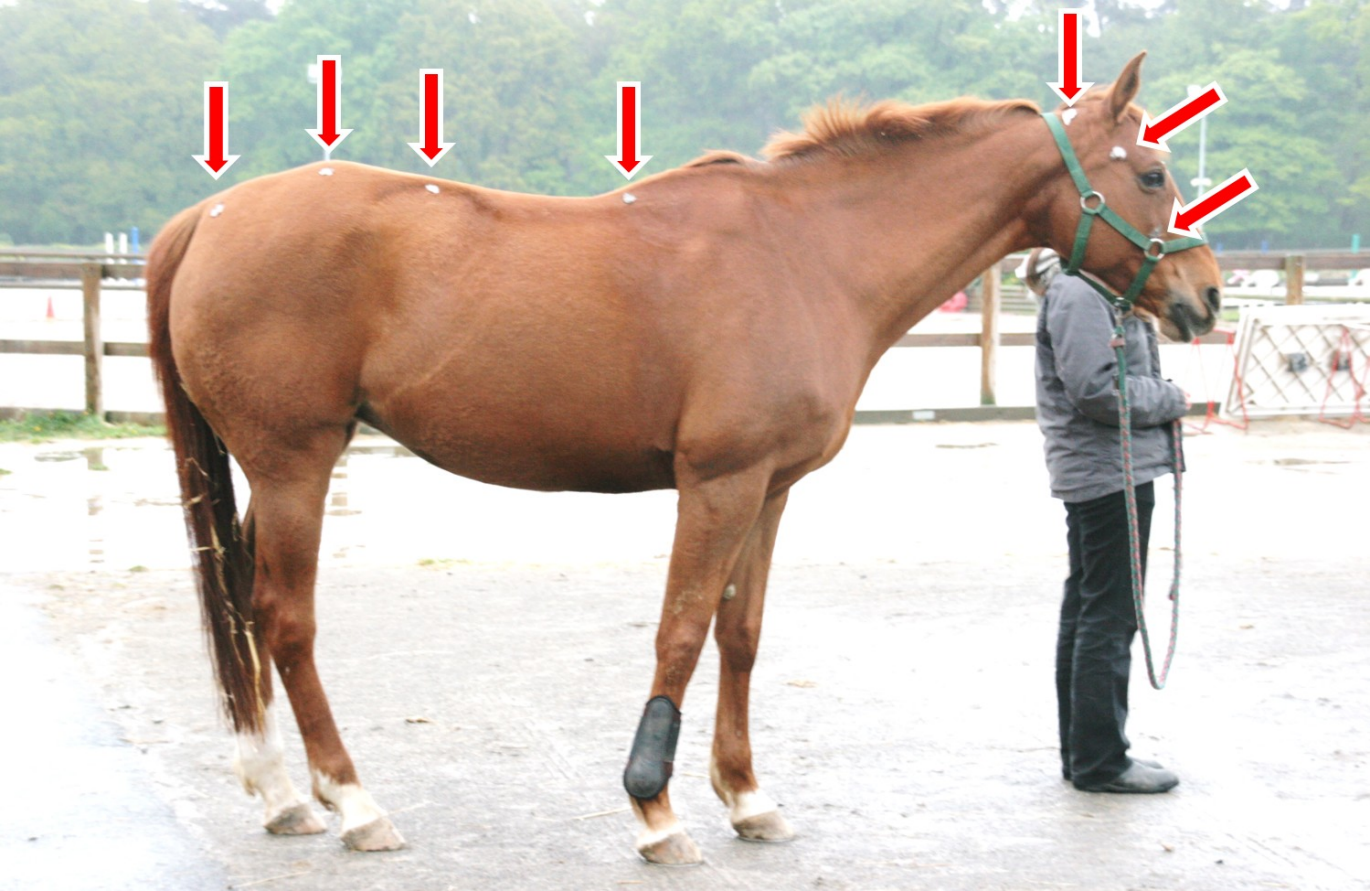
Location of the seven grey clay marks on the horse body, corresponding to future landmarks.

In order to locate anatomical points later on the photographs (future landmarks), seven marks (grey clay points, visible on all coat colors) were drawn on the horses on the side where the mane was less present (then, if required, photographs were horizontally turned in order for them to all be in the same orientation). The marks were placed on a sagittal plane (or in parasagittal plane if necessary to see the marks on the photographs) in relation to skeletal cues (thus corresponding to anatomically homologous points) from head to croup along the spine, easily identified by palpating the horse (Fig 1). Marks were placed on: the first coccygeal vertebra; the lumbo-sacral and the thoraco-lumbar junctions; the tenth thoracic vertebra (corresponding to the lower point of the withers); the atlas; the temporo-mandibular joint; the rostral extremity of the facial crest (Fig 1).

Horses were photographed both while walking (a usual situation for horses, which can provide spontaneous postures) and standing motionless near an unfamiliar experimenter (a convenient situation to take photographs). The experimenter did not talk to the horse, stayed on its left side, with a slack rope, at a predefined distance from the horse’s head (1 m), so that the experimenter never pulled the rope or the horse’s head (Fig 1). Horses were free to stand still and hold their head and neck as they wanted. Horse postures were recorded using photographs taken by another experimenter perpendicularly 10±1 m from the horse (digital camera Canon EOS 20D, zoom lens 50 mm to limit perspective distortions). Photographs were made on a regular ground, in a quiet environment (i.e. outside working time in the facility).

Preliminary simulations (bootstraps) on another data set had shown that a number of 10 photographs while standing motionless near an experimenter, and 20 photographs while hand walking, was sufficient to take into account the intra-individual variability. The median number of photos per individual while standing motionless was 10 (which was also equal to the first and third quartiles), with a range (minimum and maximum) of 6 to 12 respectively, whereas the median number of photos per individual while hand walking was 20 (like the first and third quartiles), with a range (minimum and maximum) of 14 and 32 respectively.

### Geometric morphometric treatment

**Fig 2:**
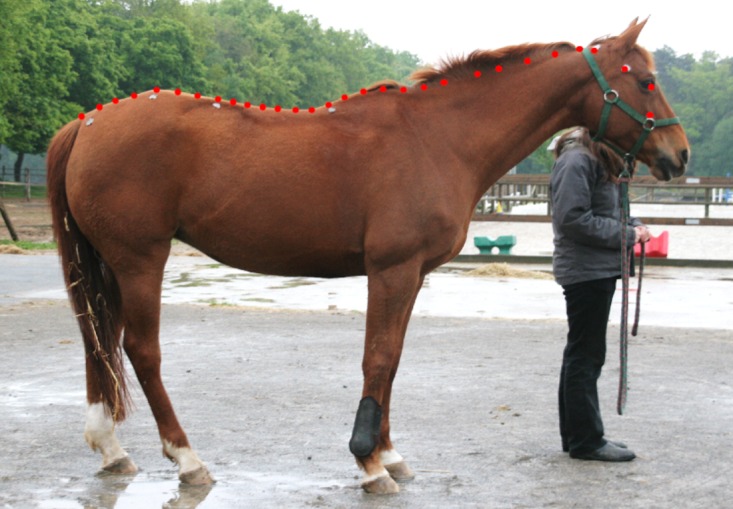
Location of the thirty semilandmarks before sliding.

Thirty landmarks (LD) were digitized by only one experimenter (ES, previously trained to use this specific set of landmarks) from the photographs using tpsDig2 software (tps software are available on http://life.bio.sunysb.edu/morph/). Their location is shown on Fig 2. The files were then loaded from tpsDig2 into tpsUtil to be combined in a single file.

This file was then loaded into R (version 3.1.2, The R Foundation for Statistical Computing, http://www.r-project.org/foundation/) to create, if necessary, sliding semilandmarks (SSL) and start shape analysis (R libraries: *ade4*, *geomorph*). SSL were defined according to the approach minimizing the bending energy [28]. Generalized Procrustes analysis and principal component Analysis (PCA) on the Procrustes coordinates were then conducted to visualize the distribution of the shape configurations corresponding to horse postures. Graphic representations of the PCA were performed thanks to the *ade4* library (version 1.7–2). Deformations corresponding to each principal component of the PCA can be visualized thanks to deformations grids created using the *geomorph* library (version 2.1.6).

Each digitized landmark can be treated as such or turned into SSL. Three geometric morphometric methods of shape analysis were tested: 1) The first method (landmarks method) corresponded to the previous study on horses [1]: 9 LD were studied. The 7 marks drawn on each horse were used in addition to the medial canthus of the eye and the middle of the neck upline (between the atlanto-occipital joint and the base of the withers), corresponding approximately to the nuchal ligament (just under the mane). 2) The second method (mixed method), used the 7 marks and the median canthus of the eye as LD as well as the 22 other points drawn on the upper midline of the horses are defined as SSL. 3) The third method (SSL method) used just the median canthus of the eye as LD while the 29 others points were defined as SSL (Fig 2).

The object of the mixed method is to draw curves using SSL, while keeping anatomical information thanks to the LD. The purpose of the SSL method is to limit errors of LD positioning as much as possible.

**Fig 3:**
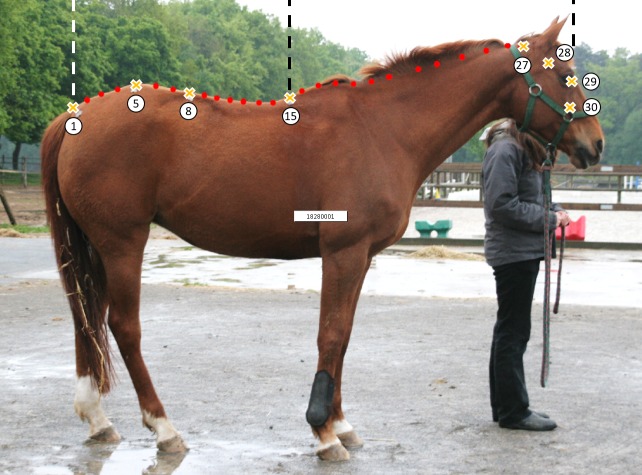
Location of the different points used for the mixed method. The landmarks are represented as crosses and the SSL as red points, and points used for the study of parts of the dorsal outline.

These three methods can be applied to the dorsal midline of the horse, or just on sections of it in order to study if some portions are more informative than others. By deleting some LD or SSL, we can focus on back and croup only (points 1 to 15, Fig 3), or on neck and head only (points 15 to 30).

Eliminating the rotation of an angle between two sets of landmarks requires defining three points: one represent the vertex of the defined angle and the two others points are placed on each set of landmarks respectively. Depending on the two points chosen on the two sets of landmarks, the fixed angle is not exactly the same and the Pinocchio effect is more or less reduced. Thus several attempts to eliminate the neck rotation were made in order to discover which better minimized the Pinocchio effect.

### Statistical analysis

The effects of the riding schools parameter and identity paramters were studied on the first three principal components (abbreviated PC) resulting from the Principal Component Analysis (PCA) based on the Procrustes coordinates through mixed model Analyses of Variance (ANOVA) where individuals were considered as a random factor. The F-statistic values resulting from the ANOVAs were extracted to compare the effect of one parameter between the different methods, and the p-values to determine the impact of the parameters.

The statistical analyses and graphic illustrations were performed with R version 3.1.2, using the *geomorph* (version 2.1.6), *ade4* (version 1.7–2) and *lme4* (version 1.1.18) libraries. The level of significance of all the statistical tests was set at 5%.

## Results

The types of populations differed somewhat between riding schools, with some having mostly horses (*e*.*g*. riding schools 1 and 2) and others (*e*.*g*. riding schools 4 and 8) having mostly ponies (chi-squared, p = 0,018). Most riding schools presented a majority of individuals with mesomorphic proportions, except schools 1 (brachymorphic only), 7 (as many mesomorphic as brachymorphic) and 3 (nearly as many number of each proportions) (chi-squared, p < 0,001). There were no differences between riding schools in terms of horses’ age or sex (respectively Kruskal-Wallis, p = 0,075; and chi-squared, p = 0,524).

For all PCAs, the contribution of the first principal components (PC) varied between 32,7% (with the mixed method on neck and head when hand walking) and 68,5% (with the SSL method on the dorsum when standing motionless); PCs2 varied between 10,8% (with the SSL method on the dorsum when standing motionless) and 35,8% of variance (with the SSL method on the croup and back, when standing motionless); PCs3 varied between 7,1% (with the SSL method on the dorsum when hand walking) and 20,2% of variance (with the mixed method on the neck and head, when hand walking) (Table 1). PCs4 were discarded because of a percentage of variability less than 10%.

**Table 1:** Percentage of variance of the third first principal component of the landmarks configuration PCA of each method and approach (PC = principal component).

	Standing motionless			Hand walking		
GM method	PC 1	PC 2	PC 3	PC 1	PC 2	PC 3
**Landmarks**	46,4	17,5	10,2	44,8	18,3	12,0
**SSL :**						
Dorsum	68,5	10,8	7,4	67,2	12,8	7,1
Dorsum without neck rotation	48,4	15,4	11,4	42,6	19,5	14,5
Croup + back	39,3	35,8	11,9	43,2	34,7	10,9
Neck + head	49,7	22,4	15,0	46,5	27,7	12,9
**Mixed:**						
Dorsum	55,0	15,6	10,0	53,2	16,0	11,8
Dorsum without neck rotation	46,3	16,1	11,1	41,9	21,0	11,1
Croup + back	57,8	24,4	10,7	55,3	20,7	11,6
Neck + head	39,8	23,9	16,2	32,7	24,4	20,2

**Fig 4:**
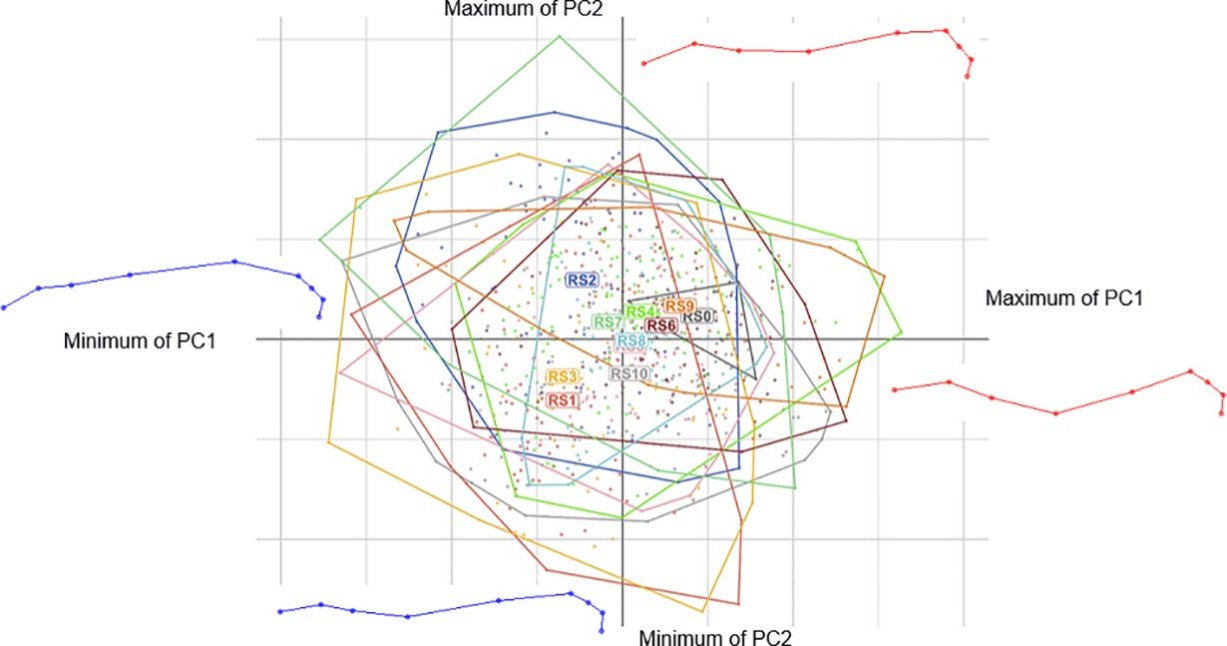
Results of the first two dimensions of the principal components analysis performed on the GLS with the landmarks method, for ‘standing motionless’. RS = riding school. The deformation grids corresponding to each extremum of the two PCs are represented (maximum in red, minimum in blue). In order to better compare the deformation grids, they are superimposed on the other figures.

Fig 4 explains how to read the superimposition of deformation grids on the following figures. Each deformation grid corresponds to the extremum (maximum or minimum) of deformation associated with a given PC. Red grids correspond to the maximum deformation of the PCs; blue grids correspond to the minimum deformation of the PCs; grey grids correspond to the consensus of each PCA.

According to the results of the ANOVA, most of these approaches proved useful in optimizing the differences between riding schools (i.e. lower p-value than with the previous LD method), but the methods (SSL and mixed) on the dorsum with neck movement proved less efficient (i.e. higher or quite similar p-value than with the previous LD method) (Tables 2 and 3).

**Table 2:** ANOVA results for standing motionless on the RS (PC = principal component).

GM method	PC 1		PC 2		PC 3	
	F	p-value	F	p-value	F	p-value
**Landmarks**	3,2	<0,001	2,17	0,017	3,93	<0,001
**SSL :**						
Dorsum	2,84	0,002	2,07	0,023	2,18	0,016
Dorsum without neck rotation	3,83	<0,001	2,67	0,003	1,14	0,325
Croup + back	2,58	0,004	4,16	<0,001	2,57	0,004
Neck + head	2,22	0,014	3,57	<0,001	2,52	0,005
**Mixed:**						
Dorsum	3,39	<0,001	1,94	0,036	4,79	<0,001
Dorsum without neck rotation	4,56	<0,001	3,48	<0,001	5,15	<0,001
Croup + back	12,7	<0,001	4,05	<0,001	2,23	0,014
Neck + head	4,07	<0,001	2,63	0,003	1,97	0,0326

**Table 3:** ANOVA results for hand walking on the RS (PC = principal component).

GM method	PC 1		PC 2		PC 3	
	F	p-value	F	p-value	F	p-value
**Landmarks**	2,63	0,0034	2,26	0,0122	5,99	<0,001
**SSL :**						
Dorsum	2,26	0,0125	2,05	0,0246	1,45	0,153
Dorsum without neck rotation	4,63	<0,001	1,58	0,107	0,797	0,632
Croup + back	4,43	<0,001	2,21	0,0148	3,89	<0,001
Neck + head	1,64	0,09	1,31	0,216	1,96	0,0328
**Mixed:**						
Dorsum	2,89	0,0013	2,16	0,017	6,83	<0,001
Dorsum without neck rotation	4,64	<0,001	7,66	<0,001	2,82	0,00171
Croup + back	13,9	<0,001	5,69	<0,001	3,74	<0,001
Neck + head	0,917	0,516	1,86	0,0461	2,3	0,0109

### Study on the dorsum

**Fig 5:**
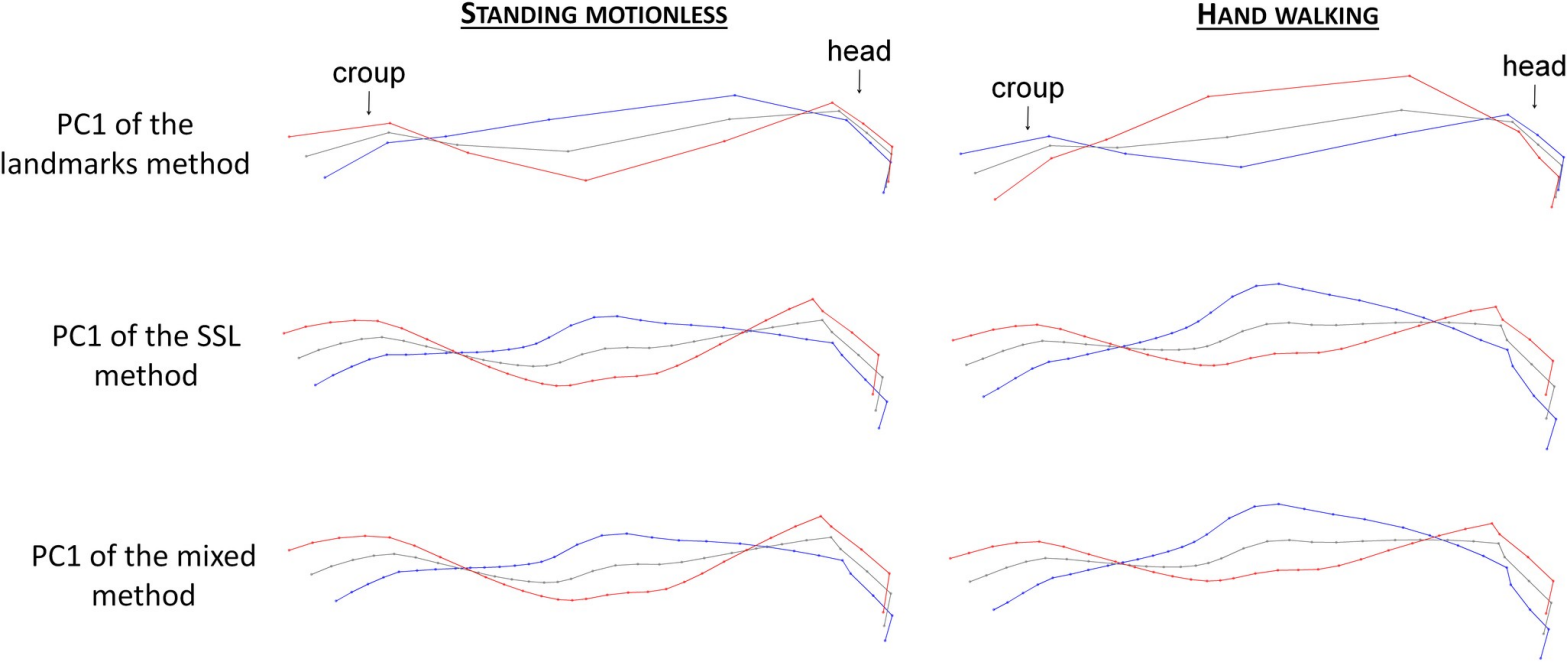
Deformation grids associated with the PC1 of the three methods on the entire upper line. Red = maximum of the PC; blue = minimum of the PC.

The first PC of these approaches, for ‘standing motionless’ as well as for ‘hand walking’ supported more than 50% of the variance, yet these principal components are mostly related to the movement of the neck (Fig 5).

The methods on the dorsum are related to identity parameters to the same extent or even more than the landmarks method (Tables 4 and 5). All three methods were highly associated with type of equid (when ‘standing motionless’ and ‘hand walking’; Figure in S1 Appendix), and to a lesser extent to the age when ‘standing motionless’. The SSL method is more associated with the proportions when ‘standing motionless’ and ‘hand walking’ (Figure in S2 Appendix**)**. The mixed method was also more related to the proportions when ‘hand walking’. Thus all first three PCs of the landmarks, mixed and SSL methods on the dorsum are linked to at least one identity parameter (type of equid, proportion or age). For further studies it would be interesting to find a method in which a part of the PCs is independent of these parameters. Nevertheless none of the methods showed difference according to sex.

**Table 4:** ANOVA results for standing motionless on the identity parameters (PC = principal component).

GM method\ Identity parameter		Type of Equid		Proportions		Age		Sex	
		F	p-value	F	p-value	F	p-value	F	p-value
**Landmarks**	PC1	18,5	**<0,001**	0.90	0,408	4,84	**0,028**	0,82	0,366
	PC2	3,48	0,062	3,61	**0,027**	1,06	0,303	<0,01	0,964
	PC3	12	**<0,001**	0,15	**0,859**	0,03	0,87	0,66	0,418
**SSL :**									
Dorsum	PC1	17,7	**<0,001**	1,94	0,144	3,84	**0,05**	0,63	0,427
	PC2	6,27	**0,012**	4,68	**0,001**	1,26	0,262	1,92	0,166
	PC3	1,08	0,298	0,38	0,682	7,36	**0,007**	0,61	0,434
Dorsum without neck rotation	PC1	27,7	**<0,001**	4,42	**0,012**	0,45	0,5	0,16	0,683
	PC2	<0,01	0,932	2,71	0,067	0,19	0,655	1,8	0,179
	PC3	0,04	0,849	0,04	0,959	8,24	**0,004**	5,75	**0,017**
Croup + back	PC1	2,16	0,142	4,44	**0,012**	<0,01	0,928	0,05	0,826
	PC2	0,04	0,84	3,82	**0,022**	<0,01	0,935	0,97	0,321
	PC3	4,68	**0,031**	1,75	0,174	2,63	0,105	0,77	0,378
Neck + head	PC1	2,09	0,148	4,64	**0,009**	2,6	0,107	0,05	0,822
	PC2	7,41	**0,006**	1,66	0,19	1,31	0,252	2,53	0,112
	PC3	19,6	**<0,001**	2,59	0,075	1,06	0,303	0,08	0,784
**Mixed:**									
Dorsum	PC1	23,4	**<0,001**	1,93	0,145	4	**0,045**	0,66	0,417
	PC2	4,98	**0,026**	2,63	0,072	2,35	0,125	0,36	0,551
	PC3	4,74	**0,029**	1,34	0,263	0,38	0,535	0,33	0,569
Dorsum without neck rotation	PC1	30,9	**<0,001**	3,39	**0,034**	0,68	0,409	0,45	0,501
	PC2	2,58	0,108	2,09	0,124	2,23	0,135	0,02	0,894
	PC3	0,08	0,774	0,76	0,469	0,04	0,847	0,34	0,562
Croup + back	PC1	0,07	0,785	1,77	0,171	2,94	0,086	0,58	0,445
	PC2	0,06	0,801	1,61	0,199	1,29	0,255	1,42	0,234
	PC3	2,32	0,128	2,55	0,078	0,186	0,666	0,01	0,917
Neck + head	PC1	13,5	**<0,001**	0,07	0,93	0,37	0,544	0,89	0,346
	PC2	22,1	**<0,001**	5,67	**0,003**	2,33	0,127	0,16	0,688
	PC3	<0,01	0,956	2,17	0,114	0,45	0,504	2,22	0,136

**Table 5:** ANOVA results for hand walking on the identity parameters (PC = principal component).

GM method\ Identity parameter		Type of Equid		Proportions		Age		Sex	
		F	p-value	F	p-value	F	p-value	F	p-value
**Landmarks**	PC1	4,36	**0,036**	0,28	0,754	1,74	0,187	0,02	0,894
	PC2	1,39	2,238	2,17	0,114	0,64	0,423	0,06	0,808
	PC3	10,4	**0,001**	0,1	0,908	1,24	0,266	0,3	0,586
**SSL :**									
Dorsum	PC1	5,31	**0,021**	0,44	0,646	1,2	0,273	<0,01	0,98
	PC2	3,11	0,078	3,54	**0,03**	0,03	0,856	3,07	0,08
	PC3	2,62	0,106	0,43	0,65	2,93	0,087	1,45	0,228
Dorsum without neck rotation	PC1	13,3	**<0,001**	2,37	0,093	<0,01	0,932	0,37	0,544
	PC2	0,5	0,479	1,63	0,195	1,02	0,313	2,14	0,143
	PC3	1,78	0,182	1,34	0,262	0,82	0,366	1,7	0,192
Croup + back	PC1	0,33	0,504	1,91	0,148	1	0,318	0,2	0,656
	PC2	2,56	0,109	8,46	**<0,001**	<0,01	0,974	0,02	0,88
	PC3	4,03	**0,045**	1,39	0,248	3,98	**0,046**	1,76	0,184
Neck + head	PC1	0,05	0,826	1,44	0,237	1,12	0,29	0,55	0,457
	PC2	0,44	0,507	1,07	0,344	0,05	0,827	1,04	0,308
	PC3	17,9	**<0,001**	2,27	0,103	0,63	0,428	3,1	0,079
**Mixed:**									
Dorsum	PC1	7,73	**0,005**	0,32	0,724	1,54	0,215	<0,01	0,951
	PC2	3,39	0,065	3,48	**0,031**	1,7	0,192	0,73	0,393
	PC3	6,27	**0,012**	0,11	0,897	2,25	0,134	0,31	0,579
Dorsum without neck rotation	PC1	17,5	**<0,001**	1,77	0,171	0,07	0,799	0,22	0,639
	PC2	2,17	0,141	1,32	0,268	3,8	0,051	0,02	0,892
	PC3	0,74	0,389	0,24	0,79	1,87	0,172	0,26	0,61
Croup + back	PC1	0,02	0,877	1,5	0,223	3,4	0,065	0,14	0,712
	PC2	0,04	0,848	2,66	0,07	0,85	0,357	0,64	0,425
	PC3	2,94	0,086	1,67	0,188	1,41	0,235	<0,01	0,95
Neck + head	PC1	4,15	**0,042**	1,69	0,184	0,45	0,502	<0,01	0,944
	PC2	1,41	0,235	1,24	0,289	0,7	0,404	1,86	0,173
	PC3	6,42	**0,011**	1,47	0,23	0,65	0,42	0,02	0,902

### Dorsum without neck rotation

Several attempts to eliminate the neck rotation have permitted us to determine that fastening the angle drawn up by the number 1, 15 and 30 SSL allowed us to better minimize the Pinocchio effect.

Invalidating the movements of the neck led to increasing differentiation of the RS, at least in the first principal component for ‘standing motionless’ and ‘hand walking’ (Tables 2 and 3). For ‘standing motionless’, SSL and mixed methods showed better results for the first two principal components than the landmarks method, but only the mixed method was more sensitive on the first two principal components than the landmarks method for ‘hand walking’ (Table 2).

**Fig 6:**
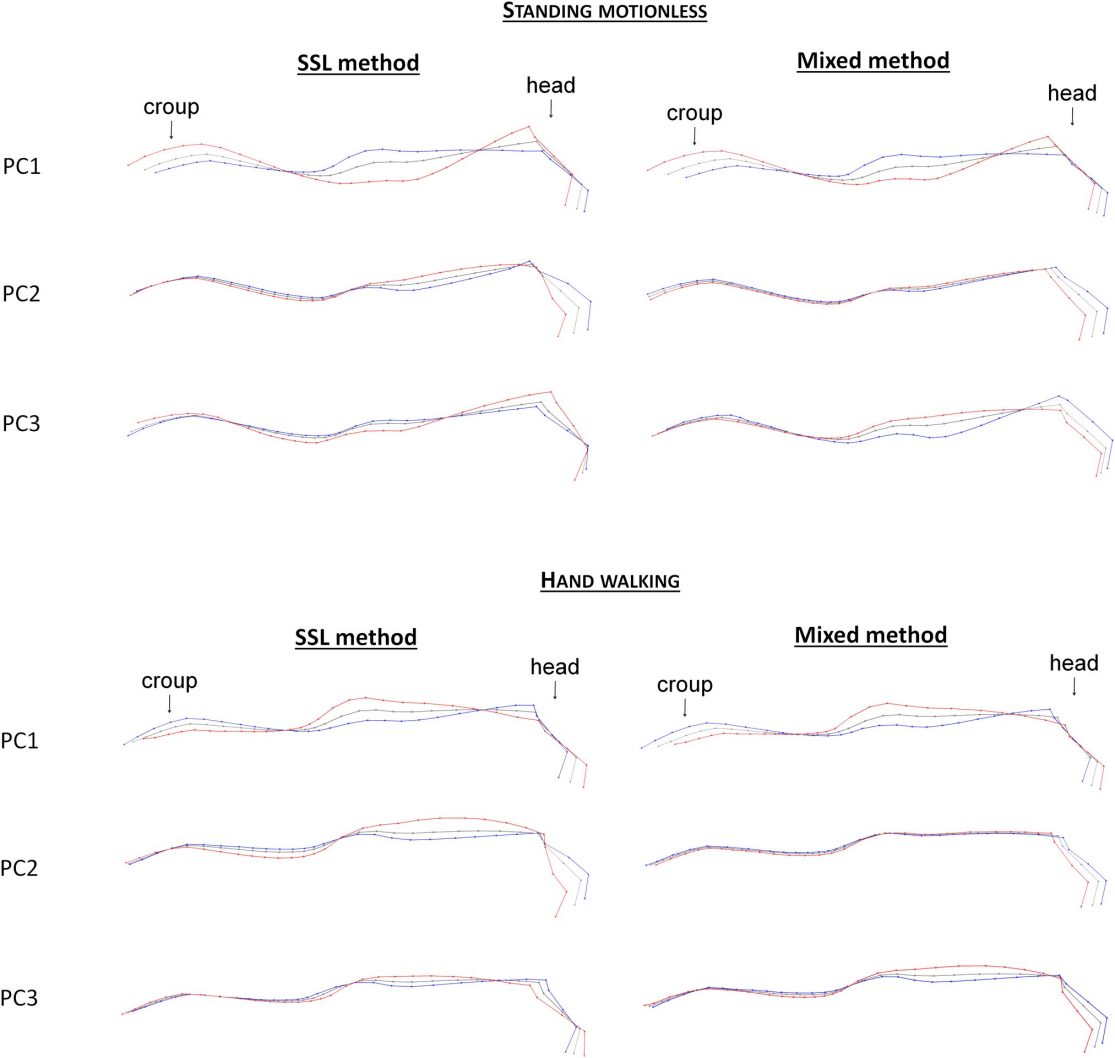
Deformation grids associated with the first three PCs of the SSL and mixed methods on the entire upper line without neck rotation. Red = maximum of the PC; blue = minimum of the PC.

The main deformations supported by the PCs1 correspond to the form and size of the croup, neck and withers, and to the angle formed by the head and the neck (Fig 6). Deformation grids corresponding to PC1 were very similar with the mixed and SSL method, either when ‘standing motionless’ or ‘hand walking’. Other PCs differently combined the variations of shape (Fig 6): e.g. PCs2 of SSL method corresponded in part to a large variation in neck roundness, whereas this element of the posture is included in PCs3 of the mixed method. Having different combinations of elements of posture appears to be useful in identifying which precise element could be linked to an intrinsic or extrinsic factor.

PCs of the mixed and SSL method on the dorsum neck rotation are less affected by identity parameters than PCs of landmarks method (Tables 4 and 5): only the PCs1 of the mixed and SSL methods were associated with the type of equids (when ‘standing motionless’ and ‘when hand walking’) and the proportions (when ‘standing motionless’); PC3 of the SSL method when ‘standing motionless’ was related to sex and age, but no correlation was found regarding the age (Pearson, r = 0,25). As some PCs are independent of identity parameters, it would be interesting to use these methods to study the impact of other factors on the posture.

### Study on neck and head or back and croup

**Fig 7:**
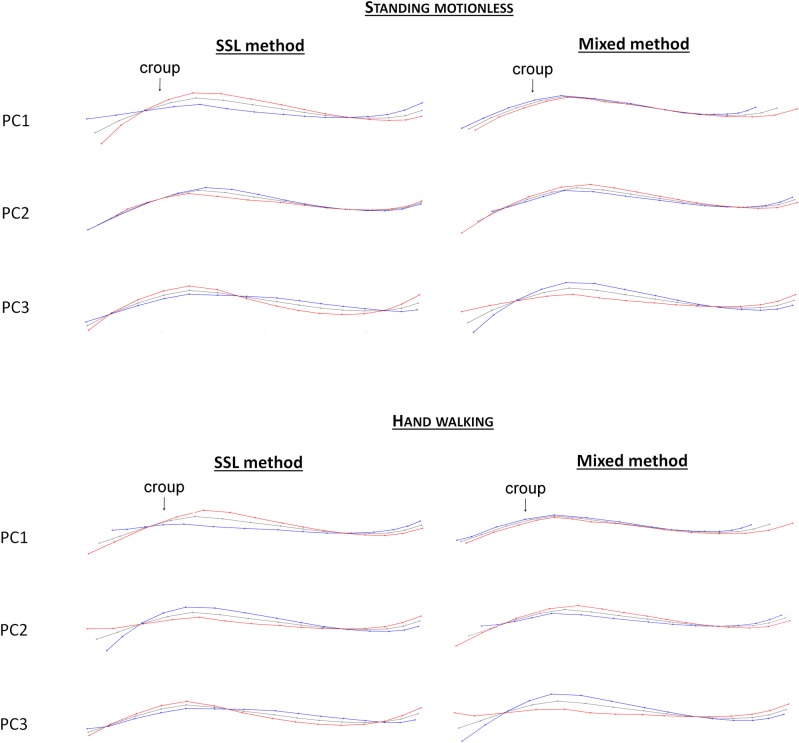
Deformation grids associated with the first three PCs of the SSL and mixed methods on the back and croup, when standing motionless and hand walking. Red = maximum of the PC; blue = minimum of the PC.

**Fig 8:**
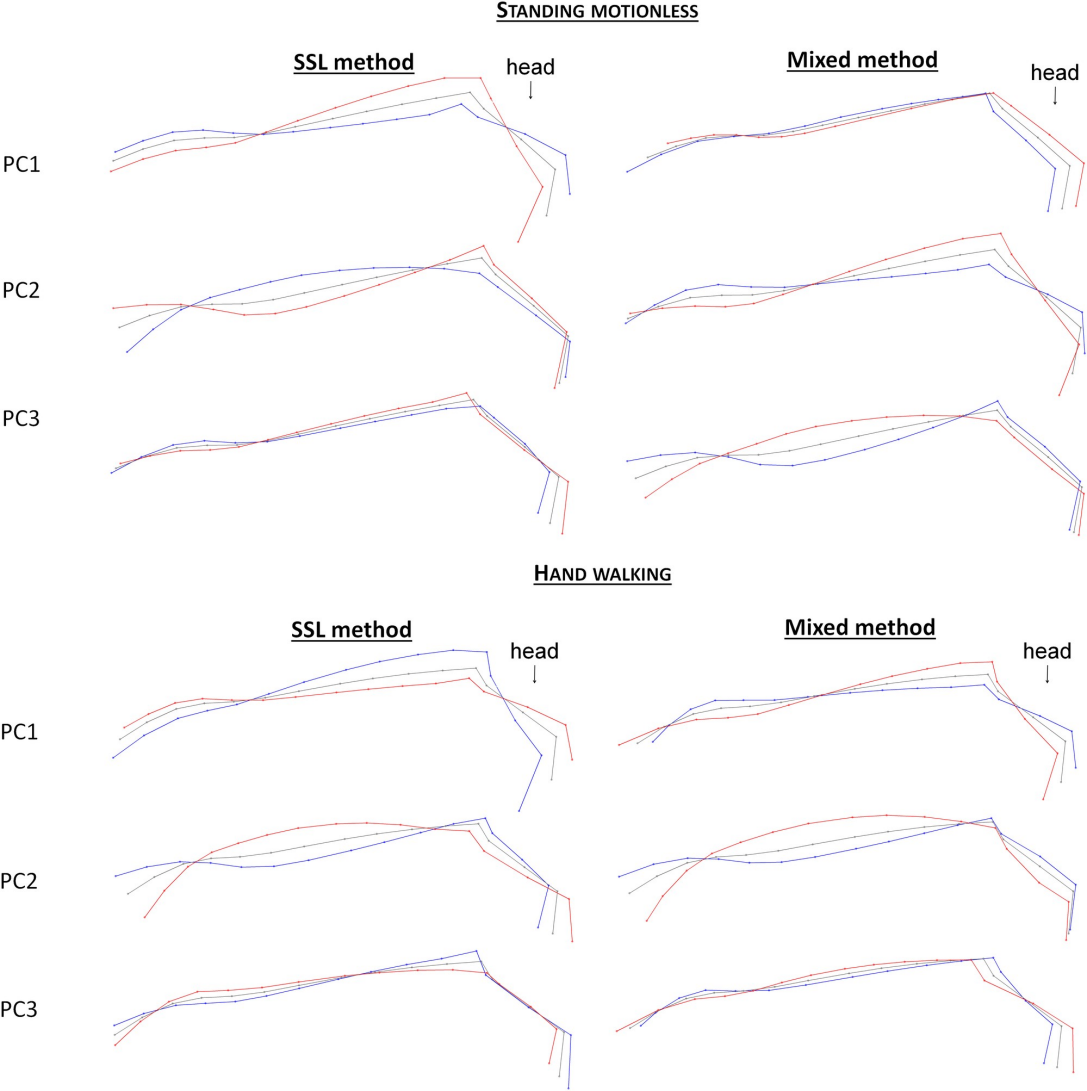
Deformation grids associated with the first three PCs of the SSL and mixed methods on the neck and head, when standing motionless and hand walking. Red = maximum of the PC; blue = minimum of the PC.

When testing an approach focusing only on a part of the upper line, it appeared that, when ‘standing motionless’, the mixed method was more effective than the landmarks method in the first two PCs in discriminating the RS. As for the SSL method, it was as efficient as the landmarks method on the first three PCs (Tables 2 and 3). These two methods provide different deformation grids (Figs 7 and 8), which combine the variations of forms or size in a different way. Therefore, retaining these two methods is interesting in order to determine which precise element of posture could be related to a given parameter.

When ‘hand walking’, only the approaches on the croup and back (as well with the mixed method as the SSL method) are more efficient than the landmarks method and can discriminate the RS on the first three PCs. Approaches on the neck and head can also distinguish the RS, but only on PC2 and/or PC3 and never on PC1. When ‘standing motionless’, each deformation grid combines differences of forms and size differently; this is promising to evaluate which elements could be informative.

Approaches on part of the upper line are, for some, related to identity parameters (Tables 4 and 5). When studying the neck and head, with the SSL method, all the PCs are associated with the type of equid (PC2 and PC3) or the proportions (PC1) when ‘standing motionless’, but none were associated when ‘hand walking’ even though deformation grids when ‘standing motionless’ and ‘hand walking’ appear quite similar (although not in detail). With the mixed method on the back and neck, there is always one PC independent of the type of equid and the proportion (PC3 when ‘standing motionless’, PC2 when ‘hand walking’). For the approaches on the back and croup, almost all the PCs of the SSL method were associated with the type of equid (PC3 as well when ‘standing motionless’ as when ‘hand walking’) or the proportions (PC1 and PC2 when ‘standing motionless, PC2 when ‘hand walking’). Moreover PC3 appeared related to age, thanks to ANOVA results when ‘hand walking’, but no correlation was found (Pearson, r = 0,19). Conversely, none of the mixed methods is linked to an identity parameter.

Similarly to the study on the dorsum without neck rotation, having some PCs independent of identity parameters could be useful to investigate the impact of other parameters on the posture.

## Discussion

As expected and proposed by [28], introducing the study of outline using SSL has allowed us to quantify and compare the horses’ upper line shapes. The use of almost only SSL, or SSL with some LD on the dorsum, led to increased riding schools discrimination.

Furthermore, as mentioned by [29], employing a fixed angle data set has implied higher statistical power (most of the p-value from the rotation-free methods are smaller) and has succeeded in highlighting changes in shape on the deformation grids that were previously hidden when the movement of neck was present.

By taking into account ANOVAs results and the variance of the principal components, the SSL and mixed method on the dorsum without neck rotation and on part of the dorsum (neck/head and back/croup) appeared the most appropriate to distinguish horse populations, whether the horse was ‘standing motionless’ or ‘hand walking’. The fact that some PCs of these approaches were independent of identity parameters and included different combinations of elements of posture could be useful to investigate the impact of other parameters on posture, like working or housing parameters, in order to find characteristic elements of posture which could be a signal of poor welfare.

Increasing the number of landmarks/SSL enabled us to describe the horse’s posture more precisely and to further reveal the importance of the roundness of the croup or the neck as markers of horse stables. We also had access to the loins and withers shape and the aspect of the tail head. This information appears in the first three PCs of several above mentioned approaches.

Interestingly, populations of individuals can be distinguished on the basis of the entire dorsal profile, as we already know (in horses: [22]; in pigs: [23]), or of the neck shape [42], but we also have observed that the back and croup shape is relevant. Several reasons can be suggested to explain this. Firstly, according to the bow and string theory (Strasser, 1913, cited by [43]), variations of head and neck posture affect the back kinematics because of the nuchal and supraspinous ligaments that connect the different segments of the upper line, from the nape of the neck to the base of the tail. Biomechanically, the head, back and neck constitute an ensemble and move together. A hollow neck and back modify the position of the pelvis and consequently the form of the croup. This hypothesis has been confirmed by kinematic studies on the effect of the head and neck position on the thoracolumbar movements (unridden horses: [54]; riding horses: [55]). Secondly, riding a horse has an impact on the back: ridden horses show a decrease of back motion [56]; inappropriate riding techniques induce a stiffness of the spine and abnormal postures, due to constant opposition of the back muscles to the actions of the rider’s hands and legs [32, 35, 57]; inexperienced riders or a poorly fitting saddle can provide an abnormal increase of the horse’s movements [58], which can be the cause of muscular spasms on the back and croup. Several studies have found variability in the prevalence of back pain or vertebral disorders among riding schools, depending on the trainer and training practices [37–39]. The authors of [38] also highlighted that clear differences appeared between schools related to the attention devoted by the teachers’ to the riders’ posture. Additionally pain, psychological stress or fear induce the dorsal muscles of the back to contract, producing extension of the spine dorsal segment which becomes visibly hollow [36]. Thus it is not surprising that the back and croup profile can change between riding schools.

Our results suggest that the neck movement was not pertinent for discriminating stables, but we can’t say if this element of horse posture is informative or not. This methodology might not be appropriate to study this particular aspect; a new methodology, such as a Procrustes analysis applied to the cyclograms of neck [59], should perhaps be developed. Movement and chronic postures are different aspects, and hence bear different information.

Optimizing the study of posture with GM was necessary as this methodology is not only inexpensive and allows use of easy statistical analysis, but also because it consists of non-invasive procedures which can be quickly applied in the field. Unlike kinematic protocols, our methodology doesn’t need costly equipment, standardized conditions (e.g. treadmill; see for review [18]) or chirurgical marker implantation which can induce pain [16]. A flat and adequately hard ground of around ten meters length is sufficient to photograph subjects directly in riding schools. Each photo shoot takes approximately ten minutes per individual. Clay marks are harmless and can be removed immediately by brushing the coat. The photo acquisition and treatment needs only a good camera and a computer.

However GM-based methodology also has several inconveniences. Firstly, the computer treatment of photographs, one by one, is rather time consuming. This step could be accelerated with an automated procedure of upper line recognition which already exists for the study of back shape in cattle for example [60]. Even though automatic recognition of the horse’s neck seems more complicated because of the mane, this could be solved with the use of marks on the base of the mane. Secondly, data treatment should be led by the same experimenter thus preventing comparisons of data taken by different experimenters in the same GLS. For the study of differences of forms not immediately visible to the naked eye (e.g. form of bones or insects wings) experimenter effect is well known, especially with the use of landmarks on rigid and unmoving structures. As far as we know this parameter wasn’t evaluated in the study of living and moving structures. As in our case the variation of forms is larger and the use of SSL enables minimization of errors of location, it would be interesting to estimate if the variation produced by different experimenters is still significant.

The new methodology allows us to go further in the study of the link between posture and identity parameters. There is no consensus in the literature on the potential influence of sex and age on the prevalence of back disorders, which can alter the horse’s posture. A previous study [39], using electromyography, found no difference according to sex nor age. In our study, ANOVA results ended with the two selected methods (mixed and SSL method on the dorsum without neck rotation and on part of the dorsum) showing significant effects of sex and age only on the PC3 of two approaches in ‘standing motionless’ and ‘hand walking’ (Tables 4 and 5). These PCs represented 11,4% (for the SSL method on the dorsum without neck rotation, when ‘standing motionless’) and 10,9% of the variance (for the SSL method on croup and back, when ‘hand walking’) (Table 1). Consequently we can assume there was a negligible effect of sex and age on the posture of riding school horses.

In agreement with previous studies [45, 46, 50], we have found a noticeable effect of the type of equid (horse or pony) and the proportions (dolichomorphic, mesomorphic and brachymorphic). Among the selected methods, only the mixed method on croup and back presents no relation to these two identity parameters. As mentioned in Material and Methods, the type of equid and proportions differ significantly between riding schools. In addition, several studies have found differences in term of welfare indicators and the prevalence of injuries between type of equid or in relation to proportions [37, 45, 49, 50]. Further investigations are needed in order to estimate to what extent postures are related to identity parameters and if other indicators (e.g. welfare indicators) could explain the observed differences between riding schools.

It was actually possible to collect an important amount of data in riding schools, including indicators of welfare, which were known to vary between schools [49]. The identification of the best methods to characterize horse postures will now allow us to examine whether postural characteristics can be related to welfare indicators and management practices (Sénèque et al., in revision).

## Supporting information

S1 Appendix Postures associated with the type of equid. Results of the first two dimensions of the Principal Components Analysis performed on the GLS with the mixed method on the dorsum without neck rotation, for ‘standing motionless’. The deformation grids corresponding to each extremum of the PC1 are represented (maximum in red, minimum in blue).
(TIF)

S2 Appendix Postures associated with the proportions. Results of the first two dimensions of the Principal Components Analysis performed on the GLS with the SSL method on the dorsum without neck rotation, for ‘standing motionless’. The deformation grids corresponding to each extremum of the PC1 are represented (maximum in red, minimum in blue). B = brachymorphic; M = mesomorphic; D = dolichomorphic.
(TIF)
