# Supplementary figures and images for: Testing optimal methods to compare horse postures using geometric morphometrics
Source: PLoS One. 2018 Oct 31;13(10):e0204208. doi: 10.1371/journal.pone.0204208 (PMC6209139; doi:10.1371/journal.pone.0204208)

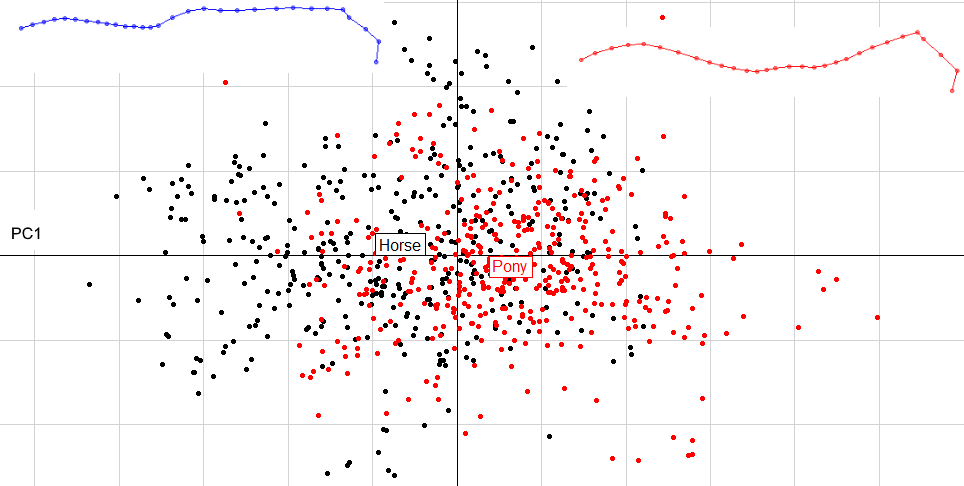

Supplement: S1 Appendix — Results of the first two dimensions of the Principal Components Analysis performed on the GLS with the mixed method on the dorsum without neck rotation, for ‘standing motionless’. The deformation grids corresponding to each extremum of the PC1 are represented (maximum in red, minimum in blue). (TIF) [file pone.0204208.s001.tif]

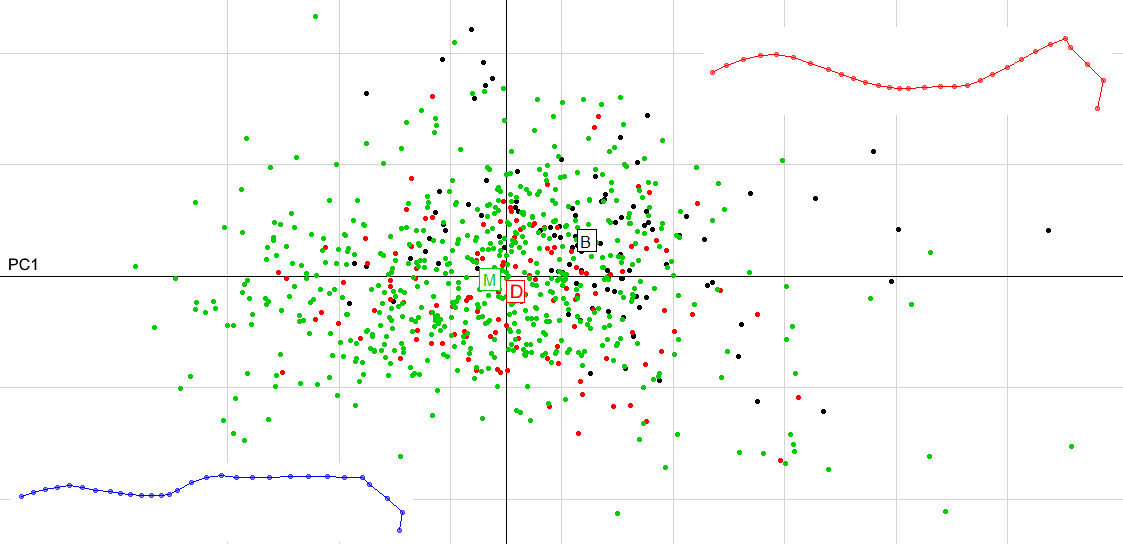

Supplement: S2 Appendix — Results of the first two dimensions of the Principal Components Analysis performed on the GLS with the SSL method on the dorsum without neck rotation, for ‘standing motionless’. The deformation grids corresponding to each extremum of the PC1 are represented (maximum in red, minimum in blue). B = brachymorphic; M = mesomorphic; D = dolichomorphic. (TIF) [file pone.0204208.s002.tif]
